# Structural Evolution of Bimetallic PtPd/CeO_2_ Methane Oxidation Catalysts Prepared by Dry Milling

**DOI:** 10.1021/acsami.1c05050

**Published:** 2021-06-02

**Authors:** Andrea Mussio, Maila Danielis, Núria J. Divins, Jordi Llorca, Sara Colussi, Alessandro Trovarelli

**Affiliations:** † Dipartimento Politecnico, Università degli Studi di Udine and INSTM, via del Cotonificio 108, 33100 Udine, Italy; ‡ Institute of Energy Technologies, Department of Chemical Engineering and Barcelona Research Center in Multiscale Science and Engineering, Universitat Politècnica de Catalunya, EEBE, Eduard Maristany 10-14, 08019 Barcelona, Spain

**Keywords:** palladium, platinum, ceria, mechanochemistry, CH_4_ combustion

## Abstract

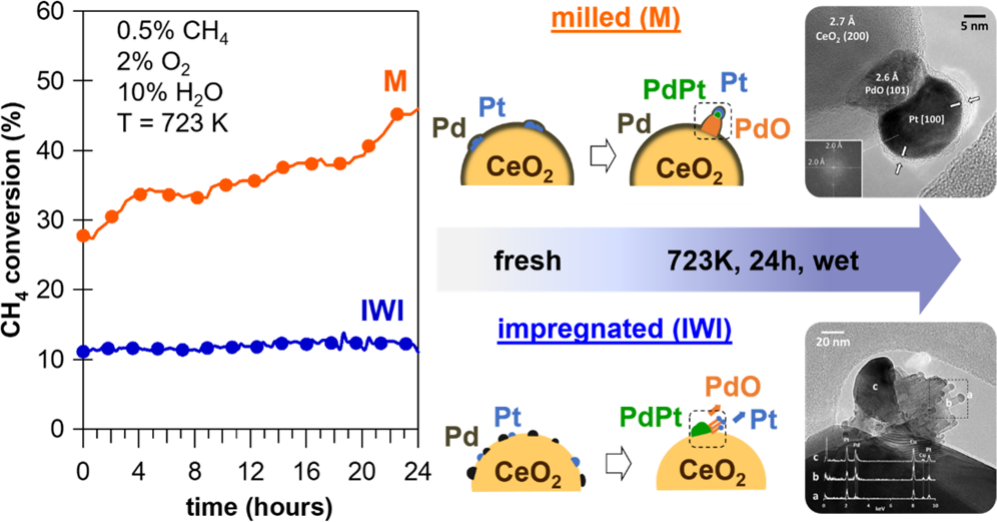

Bimetallic Pt–Pd catalysts supported on ceria have been prepared by mechanochemical synthesis and tested for lean methane oxidation in dry and wet atmosphere. Results show that the addition of platinum has a negative effect on transient light-off activity, but for Pd/Pt molar ratios between 1:1 and 8:1 an improvement during time-on-stream experiments in wet conditions is observed. The bimetallic samples undergo a complex restructuring during operation, starting from the alloying of Pt and Pd and resulting in the formation of unprecedented “mushroom-like” structures consisting of PdO bases with Pt heads as revealed by high-resolution transmission electron microscopy (HRTEM) analysis. On milled samples, these structures are well-defined and observed at the interface between palladium and ceria, whereas those on the impregnated catalyst appear less ordered and are located randomly on the surface of ceria and of large PdPt clusters. The milled catalyst prepared by first milling Pd metal and ceria followed by the addition of Pt shows better performances compared to a conventional impregnated sample and also to a sample obtained by inverting the Pd–Pt milling order. This has been ascribed to the intimate contact between Pd and CeO_2_ generated at the nanoscale during the milling process.

## 1 Introduction

The widespread diffusion of natural gas fueled vehicles (NGVs) has led to the enforcement of strict regulation on CH_4_ emissions due to the 100 year time horizon global warming potential of methane, which is 28 times that of CO_2_.^1,2^ The emission limits coupled with the low reactivity of methane molecule (least reactive among all hydrocarbons) constitute a serious challenge for methane abatement catalysts, which should attain high activity at low temperature, thermal stability, and resistance to poisoning by water and sulfur.^3^ Palladium-based catalysts are generally recognized as the most active for methane oxidation,^4,5^ but their performances are severely affected by thermal aging and the presence of steam and SO_2_ in the exhaust gases.^6−8^ Several attempts have been made to improve the resistance against deactivation, in many cases by engineering the catalyst structure^9−11^ or by the addition of co-metals and promoters.^12−18^ Indeed, an effective way to enhance the stability and durability of Pd-based catalysts is the introduction of platinum in the catalytic formulation,^19−23^ with bimetallic PtPd-based catalysts being the state-of-the-art catalysts for lean burn NGVs.^3^

The significant amount of research on PtPd bimetallic systems has clearly established the beneficial role of Pt introduction on the stability of Pd-based catalysts during time-on-stream operation and against poisoning,^12,19,23−26^ whereas the effect on the transient activity during light-off experiments is still debated, with some authors observing an improvement for specific Pt/Pd ratios^27−31^ and others concluding that the presence of Pt is generally detrimental for transient operation.^12,19,32^ The discrepancies often arise from different experimental conditions and/or catalyst pretreatment before testing. A lot of attention has been paid preferentially to the mutual interplay between palladium and platinum, which seems to be the key to tuning the catalytic performances of PdPt bimetallic catalysts,^19,23,33−36^ whereas the effect of support has been seldom considered. Nevertheless, it is well known that the activity of Pd-based catalysts for methane oxidation is highly dependent upon the metal–support interaction, particularly for the Pd–ceria systems.^37^

We have recently reported the outstanding activity for methane oxidation observed on Pd/CeO_2_ catalysts prepared by mechanochemical synthesis, which has been attributed to the strong palladium–ceria interface interaction obtained at the nanoscale during the milling of Pd and CeO_2_ powders, resulting in a mixture of Pd^0^/Pd^2+^ on the outermost layer of the catalyst particles.^38,39^ This solvent-free preparation method determines the type and distribution of the palladium-active species on the surface, which are substantially different from those obtained by the conventional incipient wetness impregnation.^39^ The idea was then to exploit the potential of the mechanochemical route to prepare bimetallic PdPt catalysts with enhanced metal–metal interaction, which could improve the stability of milled Pd/CeO_2_ in the presence of water. In this work, Pt black nanopowder is mechanically supported on ceria together with palladium with different Pt/Pd ratios. The obtained catalysts are tested for the lean methane oxidation in transient and in time-on-stream experiments, both in dry and wet (10 vol % H_2_O) atmosphere, and characterized by X-ray diffraction (XRD) analysis, temperature-programmed reduction (TPR), and high-resolution transmission electron microscopy (HRTEM). The results unambiguously show that a deep restructuring takes place between Pd and Pt during the operation and at the same time prove the effectiveness of the mechanical milling to prepare highly stable bimetallic PtPd/CeO_2_ catalysts with unique interface characteristics.

## 2 Experimental Section

### 2.1 Catalyst Preparation

Pure cerium oxide (CeO_2_, Treibacher Industrie, AG) previously calcined at 1173 K for 3 h was used as a support for the catalysts. Metallic palladium (Pd black, Sigma-Aldrich, surface area 40 m^2^/g) and metallic platinum (Pt black, Sigma-Aldrich, ≤20 μm, surface area 33 m^2^/g) nanopowders were mechanically mixed with CeO_2_ in a Mini-mill Pulverisette 23 (Fritsch) to obtain the samples defined as milled (M), adapting a previously reported procedure.^38^ Briefly, 1 g of powder (990 mg of CeO_2_ and 10 mg of metals) was introduced in a 15 mL zirconia jar with one zirconia sphere (ball-to-powder weight ratio of 10:1). The samples were prepared in two separate steps of 10 min each, starting by milling palladium with cerium oxide (10 min milling), followed by the addition of platinum (10 min milling). The oscillation frequency was set to 15 Hz. The samples were named (1 – *x*)*Pt*-*x*PdCe M, where *x* is the wt % of Pd. The name of the catalysts highlights the addition of platinum to a “*x*PdCe M” sample. Different mass ratios *m*_Pd_/*m*_Pt_ were considered while keeping the total PGM amount constant at 1 wt %. For comparison purposes, other two bimetallic samples with *m*_Pd_/*m*_Pt_ = 1 were prepared to evaluate the effect of the milling procedure. The first sample was prepared by inverting the order of the metals in the mechanical synthesis, firstly preparing a 0.5 wt % PtCe M catalyst and then introducing 0.5 wt % of Pd (0.5Pd-*0.5Pt*Ce M). The second one was prepared by conventional incipient wetness impregnation (*0.5Pt*-0.5PdCe IWI): CeO_2_ (same as used for milled samples) was impregnated with an aqueous solution of Pd(NO_3_)_2_ (4.8% of Pd, 99,999%, Sigma-Aldrich), dried overnight at 373 K, then subsequently impregnated with a solution of tetraammineplatinum(II) nitrate (Pt(NH_3_)_4_(NO_3_)_2_, 99%, Strem Chemicals). The resulting catalyst was again dried overnight at 373 K and calcined in static air for 3 h at 1173 K. Table 1 summarizes all samples, with the corresponding name and composition.

**Table 1:** Composition, Mass, and Molar Ratios of the Samples Considered in This Work

sample name	nominal Pt wt %	nominal Pd wt %	Pd/Pt weight ratio	Pd/Pt molar ratio
*0.8Pt*-0.2PdCe M	0.8	0.2	0.25	0.46
*0.65Pt*-0.35PdCe M	0.65	0.35	0.53	0.99
*0.5Pt*-0.5PdCe M	0.5	0.5	1	1.83
*0.2Pt*-0.8PdCe M	0.2	0.8	4	7.36
0.5PdCe M		0.5		
1PdCe M		1		
*1Pt*Ce M	1			
0.5Pd-*0.5Pt*Ce M	0.5	0.5	1	1.83
*0.5Pt*-0.5PdCe IWI	0.5	0.5	1	1.83

### 2.2 Characterization

The physicochemical properties of the samples were investigated by means of Brunauer–Emmett–Teller (BET) surface area measurements and X-ray diffraction (XRD) analysis. The surface area was measured according to BET theory following N_2_ adsorption/desorption isotherms at 77 K in a Micromeritics TriStar porosimeter. XRD patterns were collected in a Philips X’pert diffractometer equipped with an X’Celerator detector using Cu Kα radiation (λ = 1.542 Å). Data were first recorded in the 2θ range of 20–100° with a step size of 0.02° and a counting time per step of 40 s. The second set of measurements was carried out in the 2θ range of 32–48° with a step size of 0.02° and a counting time per step of 320 s to get more precise information on the Pd–Pt species. Redox properties were also studied by temperature-programmed tests in reducing (temperature-programmed reduction, TPR) or oxidizing atmosphere (temperature-programmed oxidation, TPO). TPR measurements were carried out in a Micromeritics Autochem II 2920 apparatus. A U-shaped quartz reactor was loaded with 50 mg of catalyst on a quartz wool bed and brought at 193 K by pumping liquid nitrogen into the furnace. The temperature was then increased up to 1173 K at a heating rate of 10 K/min. The gas flowrate (4.5% H_2_ in N_2_) was set to 35 mL/min. A second set of TPR experiments was performed, in which the samples were pretreated in flowing air (35 mL/min) up to 623 K for 1 h prior to reduction. TPO experiments were carried out by loading 150 mg of catalyst in a quartz microreactor on a quartz wool bed. A flow of 60 mL/min of 2 vol % O_2_ in N_2_ was passed through the reactor and three heating/cooling cycles were performed from room temperature up to 1273 K at a rate of 10 K/min. The oxygen uptake and release were monitored with an online ABB Magnos 106 paramagnetic analyzer. The most interesting samples were characterized also by high-resolution transmission electron microscopy (HRTEM) and high-angle annular dark-field scanning transmission electron microscopy (HAADF-STEM) combined with energy-dispersive X-ray (EDX) analysis using a field emission gun FEI Tecnai F20 microscope at 200 kV with a point-to-point resolution of 0.19 nm.

### 2.3 Catalytic Methane Oxidation

Temperature-programmed combustion (TPC) and time-on-stream (TOS) experiments were carried out at atmospheric pressure in a quartz tubular reactor, loaded with 120 mg of catalyst powder supported on a quartz wool bed. The total flow rate was set at 180 mL/min, corresponding to a GHSV of about 180 000 h^–1^. The gas inlet composition was 0.5 vol % CH_4_ and 2 vol % O_2_ in He for dry experiments, with the addition of 10 vol % H_2_O for experiments carried out in wet atmosphere. The reactor was placed in a furnace equipped with a PID temperature control, and a K-type thermocouple was inserted close to the catalyst bed for continuous sample temperature measurement. For the tests in wet atmosphere, a high-performance liquid chromatography (HPLC) pump provided a flow of deionized water, which was then evaporated by heating tapes to obtain the additional 10 vol % of steam in the feed gas. TPC tests in dry conditions (TPC dry) consisted of two heating/cooling cycles from room temperature up to 1173 K and back, recording the outlet gas composition both during heating and cooling ramps by an online ABB Uras 14 infrared analyzer. The second light-off cycle is taken as representative to ensure a stable catalytic behavior, if not otherwise specified.

For TPC experiments (TPC wet) and for time-on-stream tests (TOS wet) in wet atmosphere, the samples were pretreated with one dry cycle up to 1173 K. In wet TPC tests, after the dry pretreatment water was fed to the reactor and two light-off cycles were carried out up to 1173 K, followed by another cycle in dry atmosphere to check the reversibility of water deactivation. The time-on-stream experiments were carried out in wet atmosphere (TOS wet) by rising the temperature up to 723 K after the dry pretreatment and keeping it constant for 24 h.

Methane conversion was calculated as
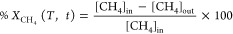
where [CH_4_]_in_ and [CH_4_]_out_ are the inlet and outlet methane concentrations, respectively. The conversion is a function of temperature during the TPC tests (the temperature ramp being constant and set at 10 K/min) and a function of time during TOS experiments at a constant temperature.

## 3 Results and Discussion

**Figure 1:**
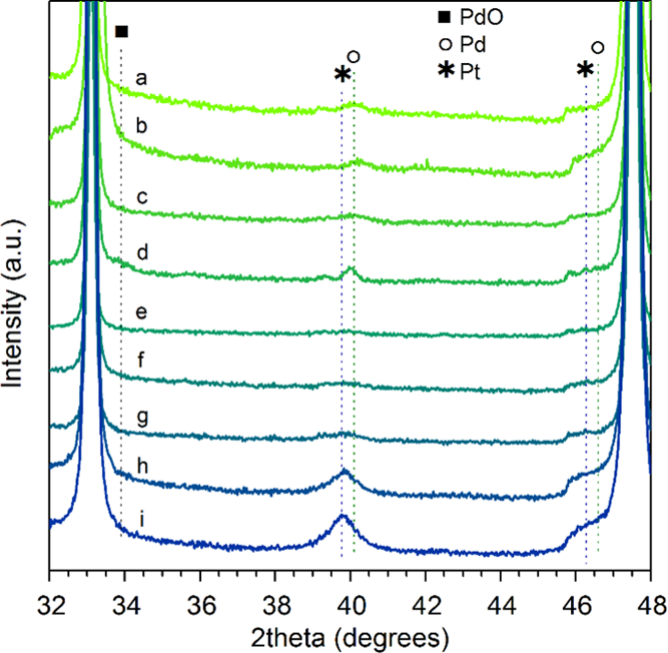
XRD profiles of fresh catalysts in the 32–48° 2θ range: (a) 1PdCe M, (b) 0.5PdCe M, (c) *0.2Pt*-0.8PdCe M, (d) *0.5Pt*-0.5PdCe IWI, (e) 0.5Pd-*0.5Pt*Ce M, (f) *0.5Pt*-0.5PdCe M, (g) *0.65Pt*-0.35PdCe M, (h) *0.8Pt*-0.2PdCe M, and (i) *1Pt*Ce M.

CeO_2_ support has a low surface area (3.0 m^2^/g) due to the high temperature of calcination (1173 K). This value is only slightly affected by the addition of the metals and the mechanical mixing process, both in the case of monometallic and bimetallic samples. For all samples, the surface area is in the range from 3.0 to 4.0 m^2^/g (see Table S1). The choice of a support with a low surface area was based on our previous studies on Pd/CeO_2_ monometallic catalysts, which showed that the best catalytic performance was achieved for the low-surface-area ceria when using Pd metal as a precursor.^39^ The X-ray diffraction profiles of the fresh samples in the 32–48° 2θ range are reported in Figure 1. Besides the characteristic peaks of cubic CeO_2_ (see also Supporting Information, Figure S1), features of Pd and/or Pt can be observed, which in some cases are barely detectable due to low metal loading. In particular, there is a visible Pt metal peak at 39.8° for the samples *1Pt*Ce M and *0.8Pt*-0.2PdCe M, together with a shoulder at 46.3°, which again corresponds to metallic Pt. On *0.5Pt*-0.5PdCe IWI also a feature at 40° is detected, which might be attributed to a PtPd alloy, as this sample is the only one subjected to thermal treatment (calcination at 1173 K). The occurrence of metallic Pd (characteristic peak at 40.1°) seems less likely since, after calcination in air, palladium is usually oxidized, as inferred from the broad shoulder at 33.9° on the same sample. As a general remark, the lattice constant of PtPd is smaller than that of Pt (3.924 Å) and larger than that of Pd (3.891 Å), so that a shift in the Pt peak to higher degrees of diffraction is expected when the alloy is formed.^40^

**Figure 2:**
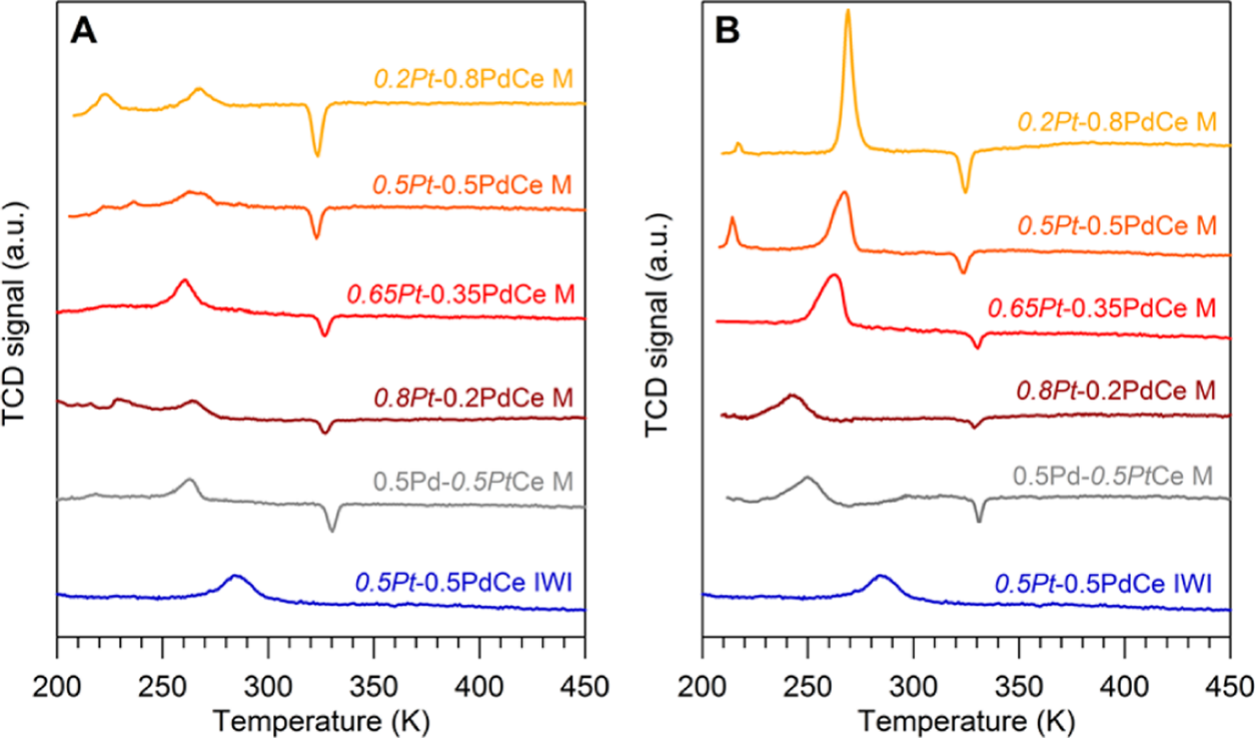
(A) TPR profiles of fresh bimetallic samples. (B) TPR profiles of bimetallic samples after the pretreatment in air at 623 K.

TPR experiments have been carried out to better understand the degree of Pt–Pd interaction on these catalysts. Figure 2a shows the TPR profiles obtained without any pretreatment, and in the case of milled samples, the hydrogen release (negative peak at about 330 K), attributed to the decomposition of Pd β-hydrides, can be observed, with the intensity increasing with increasing Pd loading. This peak can be taken as a marker of the possible alloying of Pd and Pt, as the formation of hydrides can occur only when there is free palladium on the catalyst, according to the following reactions (with *x* depending on the temperature and the H_2_ partial pressure):^41^
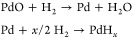
For PtPd-containing catalysts, the absence of β-hydrides peak has thus been related to the formation of an alloy between the two metals: the alloying of Pd with Pt prevents in fact its reaction with hydrogen.^42^ It is interesting to observe that this peak is not present for *0.5Pt*-0.5Pd IWI, in agreement with the X-ray diffraction data. The peaks in the lower temperature range are due to the reduction of small amounts of Pt and Pd oxides, which could be present as surface layers on the metallic powders and/or might be formed during milling. The peak at lower temperature should be attributed to the reduction of PtO*_x_*, whereas the hydrogen consumption between 260 and 270 K is due to the reduction of PdO. The attribution is made on the basis of the TPR of monometallic samples (Figure S2), even if the peaks are not so well defined, especially for Pd. If the catalysts are treated in air at 623 K before the TPR analysis (Figure 2b), the β-hydride peak at 330 K becomes slightly smaller and the hydrogen consumption at lower temperature increases for the samples containing >0.2 wt % of Pd, as expected following the Pd oxidation during the pretreatment. The quantitative analysis of the TPR data is not straightforward, as it is known that when a metal supported on ceria is reduced, it can promote the simultaneous reduction of the support at low temperature thanks to the spillover effect.^43^ This, however, does not affect the amount of hydrogen released from PdH_*x*_, which is reported in Table 2 as a calculated fraction of Pd involved. The results of Table 2 indicate that some PtPd alloying takes place after the treatment at 623 K because the percentage of PdH_*x*_ decomposition, which is proportional to the amount of free palladium, becomes slightly smaller. As a matter of fact, *0.5Pt*-0.5PdCe M shows the highest reduction in the fraction of PdH_*x*_ after the pretreatment, highlighting a higher tendency of this sample to form a PtPd alloy. The TPR profiles in the whole temperature range are reported in Figure S3.

**Table 2:** Calculated Fraction of PdH_*x*_ Decomposed during TPR Assuming *x* = 1 for All Samples

sample	%PdH_*x*_ no pretreatment	%PdH_*x*_ pretreatment @623 K
*0.8Pt*-0.2PdCe M	55	41
*0.65Pt*-0.35PdCe M	51	42
*0.5Pt*-0.5PdCe M	58	36
*0.2Pt*-0.8PdCe M	66	56
0.5Pd-*0.5Pt*Ce M	54	56

**Figure 3:**
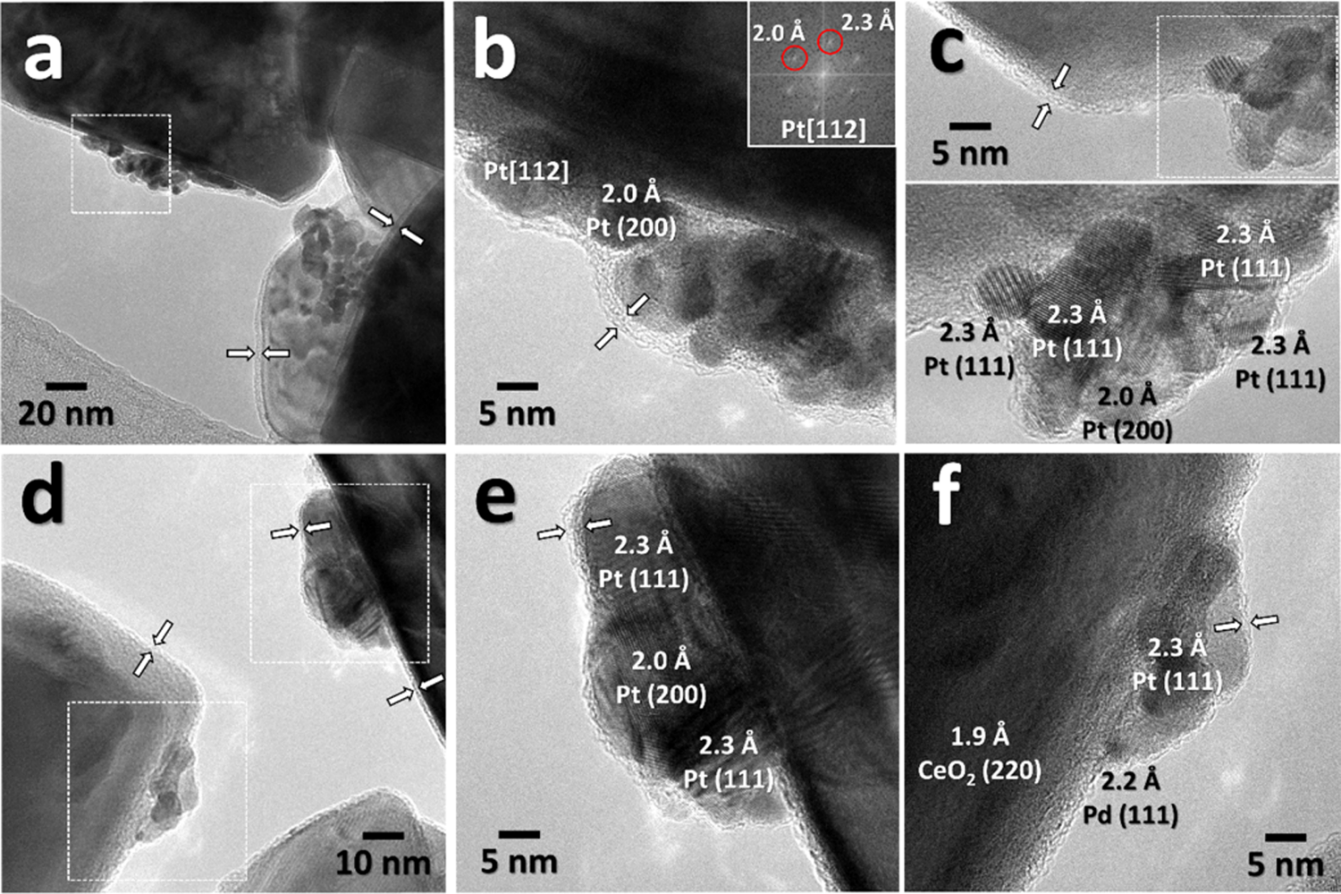
Bright-field TEM images of (a) *0.5Pt*-0.5PdCe M and (d) *0.2Pt*-0.8PdCe M with the corresponding HRTEM images of *0.5Pt*-0.5PdCe M (b, c) and *0.2Pt*-0.8PdCe M (e, f).

HRTEM was already applied successfully for the characterization of monometallic Pd/CeO_2_ catalysts prepared by dry milling, revealing the presence of a unique Pd–Ce arrangement in the form of an amorphous shell covering ceria nanoparticles (Figure S4), capable of stabilizing highly active Pd^0^/Pd^2+^ entities on the catalyst surface.^38,39,44^ HRTEM analysis of as prepared most representative bimetallic samples is shown in Figure 3. It is interesting to observe from bright-field TEM and HRTEM images that, in all cases, the addition of Pt on milled Pd/CeO_2_ does not alter the formation of the homogeneous, amorphous layer surrounding the ceria nanoparticles indicated by white arrows. The areas marked in Figure 3a,c,d are shown in the HRTEM mode in Figure 3b,c,e,f for *0.5Pt*-0.5PdCe M and *0.2Pt*-0.8PdCe M, respectively. The ceria crystallites are decorated with nanoparticles of about 10 nm and lattice fringe and Fourier transform (FT) analyses reveal that almost all of them correspond to metallic Pt (a Pd nanoparticle can be spotted on *0.2Pt*-0.8PdCe M; Figure 3f). Platinum clusters in contact with ceria are also covered by the Pd–Ce amorphous layer (Figure 3b,c sample *0.5Pt*-0.5PdCe M; Figure 3e,f sample *0.2Pt*-0.8PdCe M), somehow as if they were sliding underneath the shell during the milling process.

**Figure 4:**
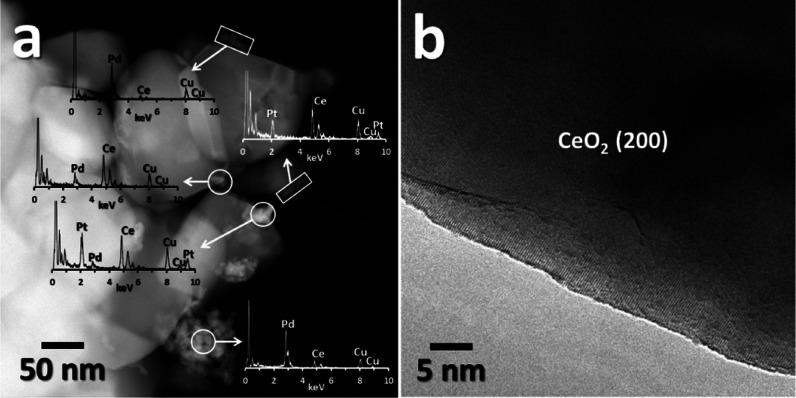
HAADF-STEM (a) and HRTEM (b) images of 0.5Pd-*0.5Pt*Ce M. The Cu signal belongs to the TEM grid.

However, this happens only when Pd is milled first with ceria, highlighting once again the uniqueness of the Pd–ceria interaction induced by the milling process. In fact, when the order of milling is inverted (i.e., first Pt with CeO_2_ followed by the milling of Pd with Pt/CeO_2_), the HAADF-STEM-EDX analysis reveals the presence of isolated Pt and Pd clusters (Figure 4a) and no amorphous layer, as shown in Figure 4b. The different interaction of Pt with ceria during milling is inferred also from the images of the monometallic Pt/CeO_2_ catalyst, where only Pt nanoparticles on ceria are observed (Figure S5). On the impregnated catalyst, Pd and Pt are detected in the form of ultrasmall nanoparticles (Figure S6).

**Figure 5:**
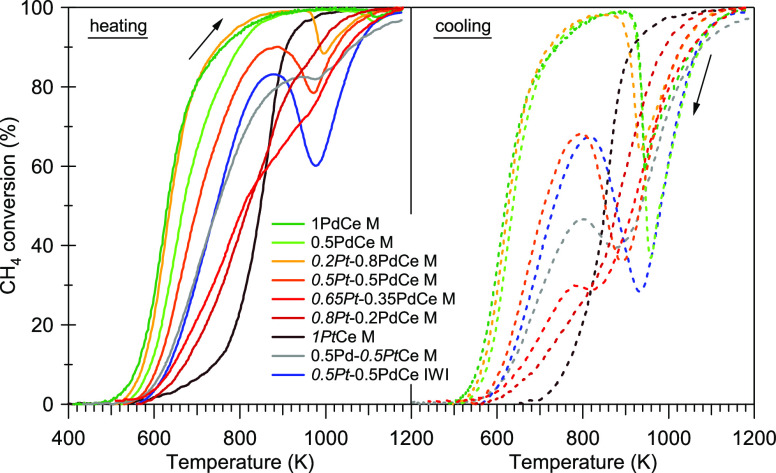
Light-off curves for methane oxidation in dry conditions (0.5 vol % CH_4_, 2 vol % O_2_ in He, GHSV ≈ 180 000 h^–1^) for all samples.

Catalytic methane oxidation tests carried out in dry conditions on bimetallic samples show that the addition of platinum negatively affects the low-temperature activity of the Pd-based samples. In Table 3, the temperatures to achieve 10% and 50% (*T*_10_ and *T*_50_) methane conversion during the heating branch of the second light-off cycle are reported, and the corresponding light-off curves are shown in Figure 5. The characteristic temperatures increase with increasing Pt loading, indicating worse light-off performances for Pt-containing samples. The situation is similar when looking at the transient light-off experiments carried out in wet atmosphere (Figure S7). The results are in line with previous literature works carried out on conventional impregnated catalysts, which in general indicate that during the light-off experiments, the samples containing Pt are less active than the monometallic Pd ones.^12,19,32,45^ Looking more in detail at the light-off curves of Figure 5, it can be observed that at high temperature, during the heating ramp, Pt has a negative impact on the redox cycle of Pd–PdO as inferred from the decrease in methane conversion, which is typically associated with the decomposition of palladium oxide.^46^ This happens because the presence of Pt anticipates the PdO decomposition to metallic Pd in a range of temperatures where the homogeneous methane oxidation is not yet self-sustaining (below 1100 K). During cooling, it is again evident that Pt changes the process of Pd re-oxidation, as indicated by the deep loss in methane conversion more pronounced for the Pt-containing samples (with the exception of *0.2Pt*-0.8PdCe M).

**Table 3:** *T*_10_ and *T*_50_ Measured during the Second Light-Off Cycle of Methane Oxidation in Dry Conditions (0.5% CH_4_, 2% O_2_ in He) for the Samples Considered in This Work

sample	*T*_10_ (K)	*T*_50_ (K)
*0.8Pt*-0.2PdCe M	670	821
*0.65Pt*-0.35PdCe M	644	810
*0.5Pt*-0.5PdCe M	609	702
*0.2Pt*-0.8PdCe M	576	637
0.5PdCe M	592	668
1PdCe M	563	631
*1Pt*Ce M	746	849
0.5Pd-*0.5Pt*Ce M	625	747
*0.5Pt*-0.5PdCe IWI	637	745

The effect is observable also from the TPO profiles recorded for 1PdCe M and *0.5Pt*-0.5PdCe M, chosen as the representative ones and reported in Figure S8. The role of Pt is clear: on one hand, it hinders the cycling of PdO-Pd-PdO (the peaks of oxygen release and uptake are barely detectable after cycle 1); on the other hand, it anticipates the PdO decomposition of about 100 K. This result was already reported in the literature, irrespective of the support.^24,28,47^

**Figure 6:**
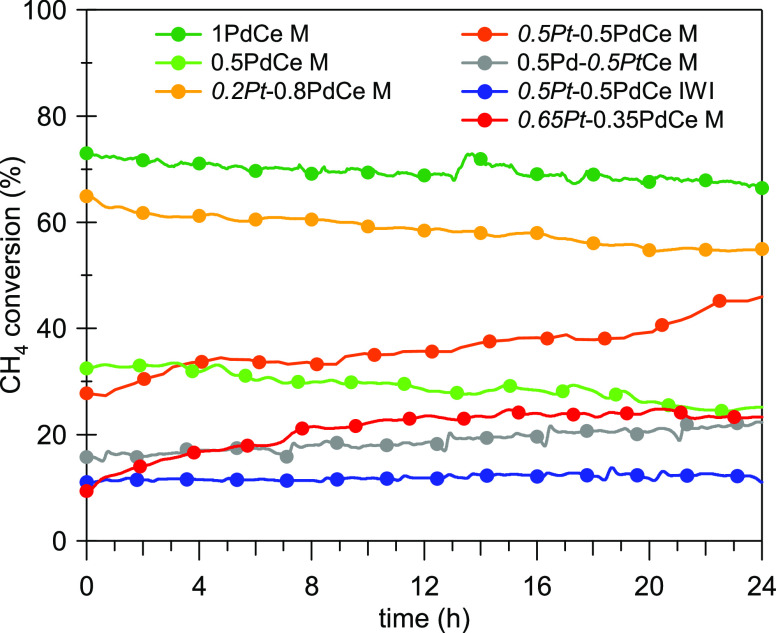
Methane conversion during TOS wet experiments (0.5% CH_4_, 2% O_2_, 10% H_2_O in He) at 723 K. Samples *0.8Pt*-0.2PdCe M and *1Pt*Ce M are not included as their activity was too low at 723 K in the presence of water.

The situation changes when considering the time-on-stream experiments carried out in wet atmosphere (0.5% CH_4_, 2% O_2_, 10% H_2_O in He) where the bimetallic milled samples with Pt content of ≥0.5 wt % showed higher stability compared to the monometallic Pd/CeO_2_ catalyst (Figure 6). For *0.5Pt*-0.5PdCe M and *0.65Pt*-0.35PdCe M, the activity increases over time. Also, during the time-on-stream experiments in the absence of water in the feed gas, the addition of platinum results in an improved catalytic activity, with a trend among different samples similar to the one observed in wet conditions (see Supporting Information, Figure S9). In the literature, this behavior has been tentatively ascribed to an enrichment in PdO and/or to a reconstruction of the surface during time-on-stream operation.^19,25,36,45^ An activation of bimetallic PtPd/Al_2_O_3_ catalysts has been reported also during repeated reduction/re-oxidation cycles, which suggests a role of the mutual redox interplay between palladium and platinum.^24^ This seems to be confirmed by the observation that after aging in air (1023 K for 10 h), only 20–30% of PdO is formed on PtPd-based samples, whereas palladium is fully oxidized (100% PdO) on the monometallic sample.^33^ The restructuring of PtPd nanoparticles has been observed also for supported Pd–Pt@CeO_2_/Si–Al_2_O_3_ catalysts, with the formation of a Pt-rich core surrounded by a Pt–Pd shell under reducing conditions.^48^

**Figure 7:**
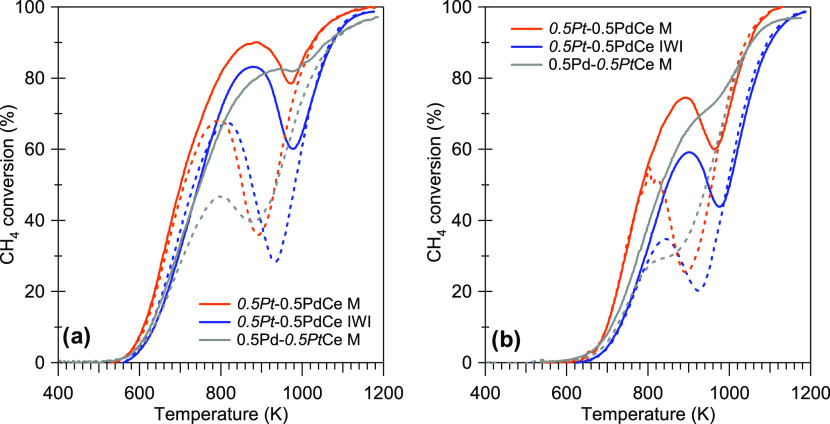
Second cycle of light-off experiments for *0.5Pt*-0.5PdCe M, *0.5Pt*-0.5PdCe IWI, and 0.5Pd-*0.5Pt*Ce M in dry (a) and wet (b) conditions. Solid line: heating. Dashed line: cooling.

It is interesting to observe that, considering the best-performing bimetallic sample, i.e., the one containing 0.5Pt and 0.5Pd, the activity of the milled sample *0.5Pt*-0.5PdCe M is much higher than that of the corresponding impregnated one, indicating that the milling procedure, already effective for monometallic samples, can be successfully applied to the preparation of bimetallic PtPd catalysts. In addition, the order of milling is also important because better results are obtained when Pd is put directly in contact with ceria, as observed by comparing 0.5Pd-*0.5Pt*Ce M with *0.5Pt*-0.5PdCe M. This is in agreement with the HRTEM evidence showing the formation of the amorphous shell only for *0.5Pt*-0.5PdCe M as a result of milling Pd and ceria first, then Pt. This result is further corroborated by the comparison of the transient light-off experiments both in dry and wet conditions reported in Figure 7a,b, respectively, which show that the bimetallic milled samples are better than the impregnated counterpart (especially in the presence of water) and that among them the catalyst obtained by milling Pd and ceria first has a much higher catalytic activity compared to that of 0.5Pd-*0.5Pt*Ce M.

**Figure 8:**
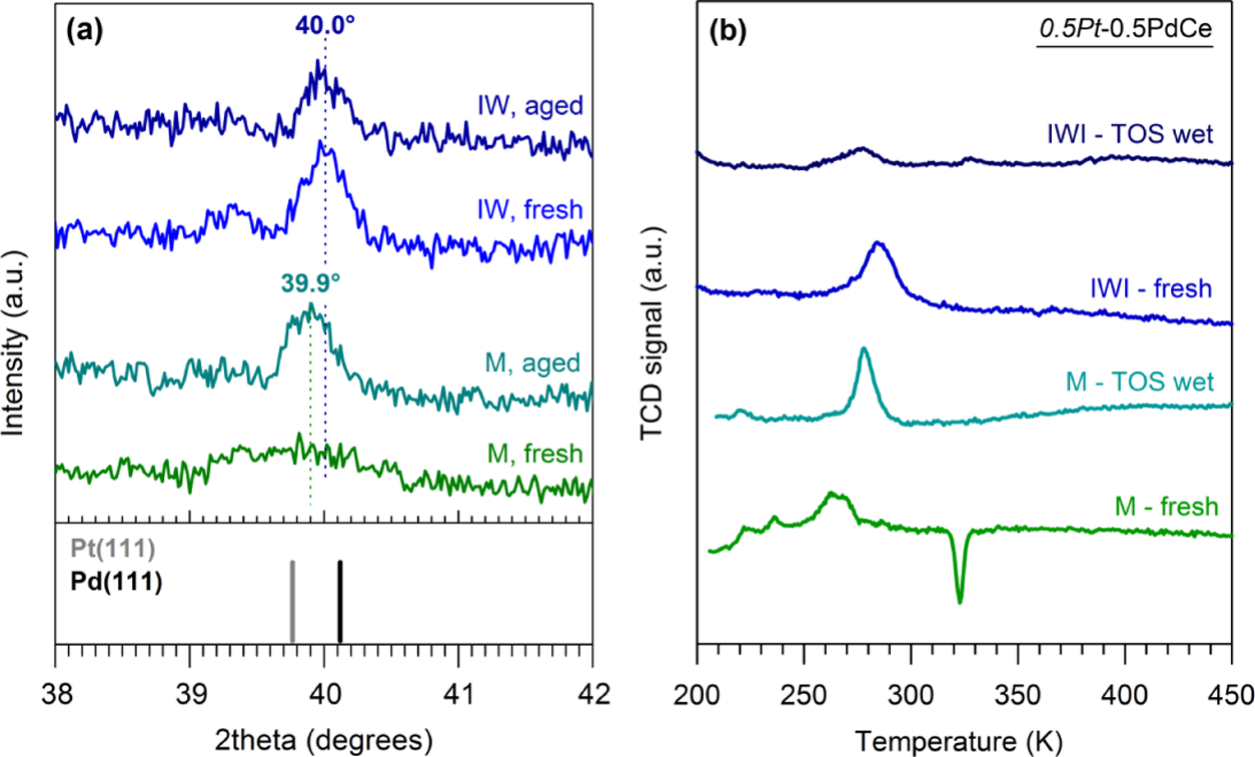
(a) XRD profiles collected on *0.5Pt*-0.5PdCe M and *0.5Pt*-0.5PdCe IWI samples, as prepared (fresh) and after time-on-stream test in wet conditions (TOS wet). Peak positions of Pd(111) (JCPDS 00-046-1043) and Pt(111) (JCPDS 00-004-0802) are reported as reference. (b) H_2_-TPR profiles of *0.5Pt*-0.5PdCe M and *0.5Pt*-0.5PdCe IWI samples, as prepared (fresh) and after time-on-stream test in wet conditions (TOS wet).

To better understand the reasons for the improved behavior of the milled bimetallic catalysts during the time-on-stream operation in wet conditions, further characterization was carried out on spent samples after TOS experiments for the most representative ones. In particular, X-ray diffraction patterns (Figure 8a) and TPR profiles (Figure 8b) show that on *0.5Pt*-0.5PdCe M, there is the formation of a PtPd alloy after the TOS treatment in wet atmosphere. This is evidenced by the feature at 2θ = 39.9°, which lies in the middle of the peaks belonging to the single metals, and by the absence, in the TPR profile, of the hydrogen release peak attributed to the decomposition of Pd β-hydrides. From the comparison of the XRD profiles of milled and impregnated catalysts, it can be observed that the TOS wet treatment has a lower impact on *0.5Pt*-0.5PdCe IWI and that on this sample the peak is shifted to the right with respect to *0.5Pt*-0.5PtCe M, indicating an alloy richer in Pd, with possibly a hint of Pd segregation.

**Figure 9:**
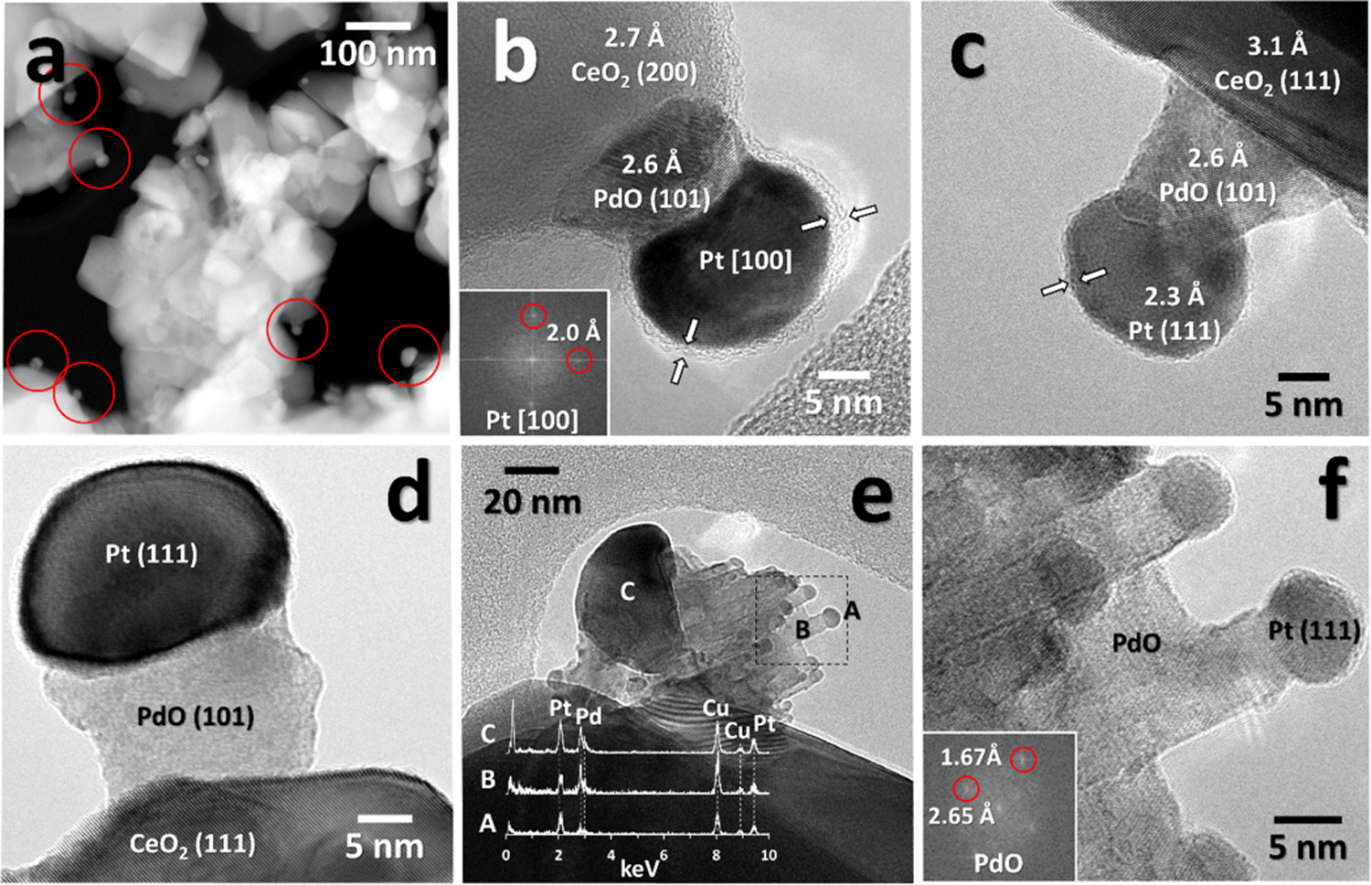
HRTEM images of the samples after TOS wet experiments: (a), (b) *0.5Pt*-0.5PdCe M, (c) *0.2Pt*-0.8PdCe M, (d) 0.5Pd-*0.5Pt*Ce M, and (e), (f) *0.5Pt*-0.5PdCe IWI.

The HRTEM characterization of the spent samples after the TOS wet (Figure 9) shows that on all milled catalysts there is a strong, unique rearrangement of platinum and palladium in the form of mushroom-like structures growing on the ceria surface. Irrespective of the milling order, the mushrooms present a PdO foot in contact with ceria supporting a Pt head more exposed to the gas phase. A similar arrangement is also seen on *0.5Pt*-0.5PdCe IWI, but with a less developed structure, where thinner, needle-like stems support a smaller Pt head (Figure 9e,f) and Pt and PdO are less clearly distinguished.

Figure 9a,b presents a general STEM-HAADF and HRTEM images of *0.5Pt*-0.5PdCe M in which the amorphous layer characteristic of the fresh milled sample is distinctly visible (indicated by white arrows) around ceria nanoparticles and PdO–Pt mushroom-like structures (some of them enclosed in circles in Figure 9a). The layer is present also on *0.2Pt*-0.8PdCe M (Figure 9c) and seems unaltered by the TOS wet treatment. Figure 9d shows the HRTEM image of the 0.5Pd-*0.5Pt*Ce M sample, in which a mushroom very similar to that observed on *0.5Pt*-0.5PdCe M is visible but without the amorphous shell, coherently with the HRTEM images of the corresponding fresh catalyst. This indicates that the formation of mushroom-like structures is not related to the presence of the amorphous Pd–Ce layer. In Figure 9e, a portion of *0.5Pt*-0.5PdCe IWI catalyst is shown, together with EDX analyses indicating the presence of big PtPd particles that seem to evolve into needle-like structures, again with a PdO stem supporting a Pt head (Figure 9f). In this case, PdO is supported on both the ceria surface and the bimetallic particles. This means that on the samples prepared by mechanical milling, the interaction between Pd and Pt has a different evolution compared to *0.5Pt*-0.5PdCe IWI, where less ordered, agglomerated structures are encountered. In contrast, the formation of very homogeneous and very well distributed PdO–Pt mushroom structures identified in the bimetallic catalysts prepared by mechanochemical synthesis is unique.

**Figure 10:**
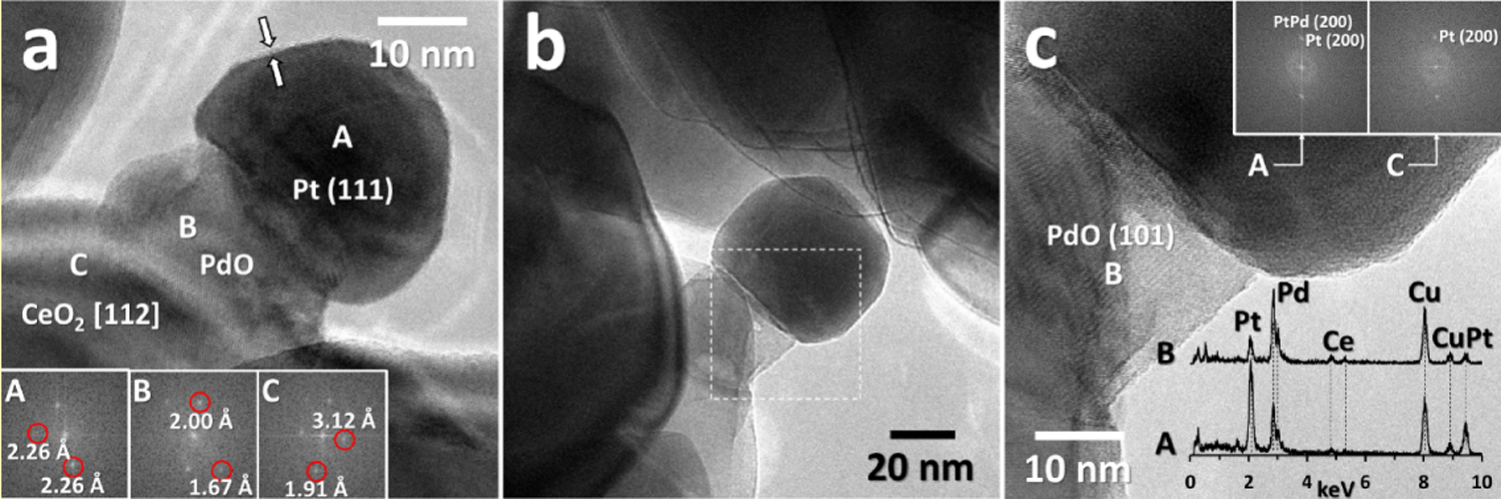
(a), (b) HRTEM images of *0.5Pt*-0.5PdCe M after one methane oxidation cycle in dry atmosphere up to 1173 K (0.5% CH_4_, 2% O_2_ in He). (c) EDX analysis and FT images of the area enclosed in the square in (b).

The improvement in activity observed during TOS wet treatment cannot be related straightforwardly to these structures because very similar ones are formed also on the samples which do not show any improvement (*0.2Pt*-0.8PdCe M; Figure 6). Moreover, similar arrangements are detected also on *0.5Pt*-0.5PdCe M after the dry methane oxidation cycle carried out up to 1173 K before the TOS experiment (Figure 10). This can indicate a general tendency of Pd–Pt clusters to evolve into these nanostructures in the reaction atmosphere.

Compared to the mushroom-like structures observed for the *0.5Pt*-0.5PdCe M sample in Figure 9a,b, Pt heads of the ones reported in Figure 10a,b show a well-faceted morphology, and overall, these structures are much bigger in size. The EDX analysis suggests the presence of PtPd bimetallic regions after the dry TPC cycle (Figure 10c), which is further corroborated by the presence of spots at 1.94–1.95 Å observed in the FT image recorded in the area labeled A, with respect to the (200) planes of Pt at 1.96 Å present in the FT image recorded in the area labeled C in Figure 10c. This supports the hypothesis of the derivation of the mushroom-like arrangements from PtPd-alloyed clusters.

**Scheme 1:**
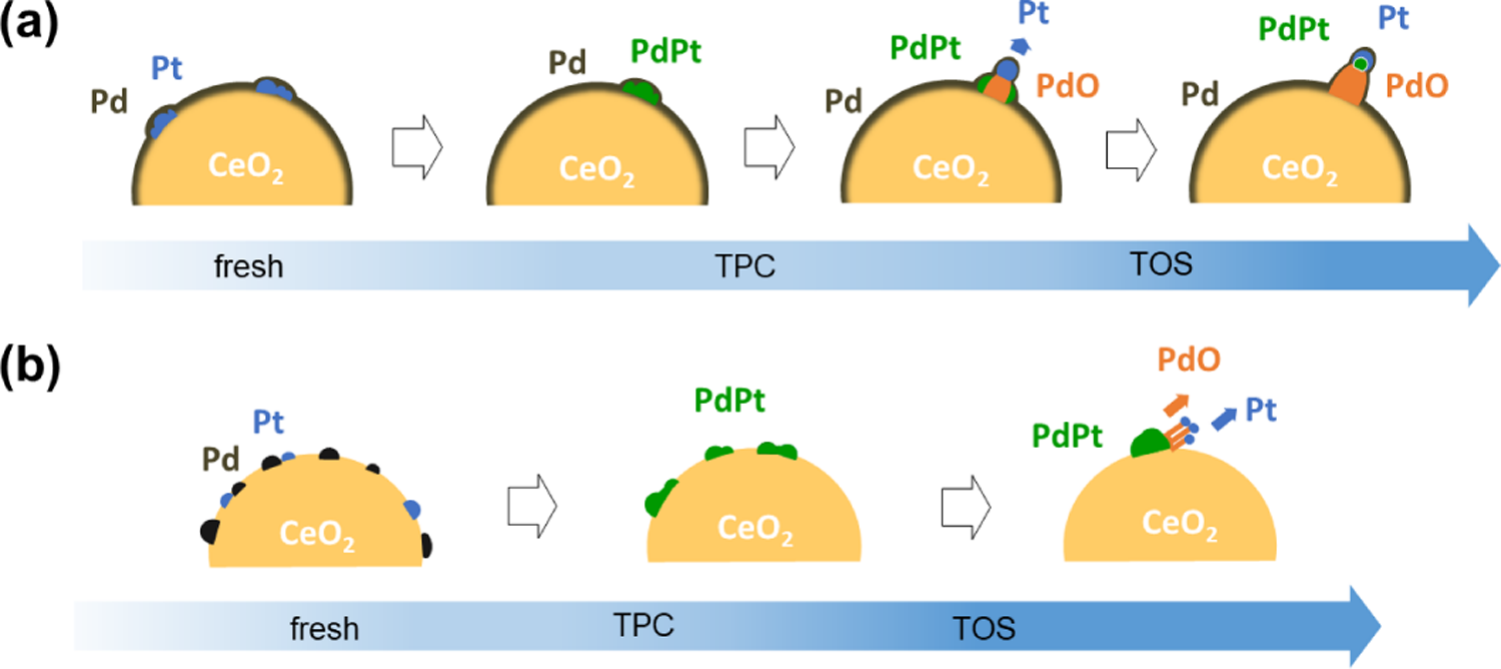
Mutual Evolution of Pt–Pd Species during TPC Dry and TOS Wet Treatments on Milled (a) and Impregnated (b) Samples

The overall picture emerging from the characterization of the spent catalysts indicates unambiguously that all samples, irrespective of the synthesis method, undergo complex restructuring, which appears to begin during the dry TPC cycle preceding the TOS wet experiment (Figure S12). This modification involves the formation of a PtPd alloy, as inferred from the XRD and TPR profiles, that on milled catalysts further evolves in the development of mushroom-like structures detected by HRTEM in which PdO and Pt nanoparticles can be observed, being, respectively, the foot and the head of the mushrooms. This evolution seems slower on the conventional impregnated catalyst, for which big PtPd clusters are still observed after TOS wet treatment with needle-like PdO–Pt arrangements growing on them. A model of the proposed mechanism for the formation of the new Pt–PdO arrangements is shown in Scheme 1.

To the best of our knowledge, this is the first time that such structures have been observed on bimetallic PtPd samples. This might be due to the fact that for the catalysts analyzed by HRTEM in previous studies, the maximum temperatures reached were lower, even though for alumina-supported samples with similar metal loading treated at 1023 K in air for 10 h only Pt–Pd and separated PdO nanoparticles could be detected.^33^ The evolution of the mushrooms could thus be linked to the effect of the ceria support, which can favor Pd oxidation as the PdO “feet” are located on the ceria surface at least for milled samples, and/or to the presence of water in the reaction atmosphere. It should be noted, in fact, that also during TPC dry treatment up to 1% H_2_O is formed as a reaction product. The effect of water though seems less likely since PtPd/Al_2_O_3_ catalysts treated at 1073 K in wet air do not show particular arrangements in TEM images.^34^ Moreover, in some papers,^30,34,35^ the formation of PdO on the surface or as a layer on the outer shell of bimetallic nanoparticles is supposed to occur, differently from what happens for the mushroom-like structures, which points again to a key role of ceria in keeping PdO anchored to its surface. Indeed, in other studies, separate PdO and PtPd bimetallic phases have been observed,^33,36^ more similarly to the impregnated catalyst, even if the occurrence of an intermetallic phase is detected also on the milled samples. However, as outlined above, this restructuring alone cannot explain the improvement in activity, which takes place predominantly on milled samples with the Pt content between 0.2 and 0.65 wt %. The evidence collected in this work indicates that the strong Pd–ceria interaction induced by the milling process, achieved only when Pd and ceria are milled first, is pivotal for both activity and stability of bimetallic PtPd-based catalysts.

## 4 Conclusions

A solvent-free mechanochemical synthesis route, developed for monometallic Pd/CeO_2_ catalysts, has been successfully applied to the preparation of bimetallic PtPd–ceria formulations, which are demonstrated to be very stable for methane oxidation in wet atmosphere, even improving their activity during time-on-stream operation for Pd/Pt molar ratios between 1:1 and 8:1. The catalyst obtained by milling first Pd and ceria then adding Pt (*0.5Pt*-0.5PdCe M) shows better performances with respect to its impregnated counterpart and also with respect to the milled sample prepared by inverting the order of milling (first Pt and ceria, then Pd). A key role of the Pd–ceria interaction at the interface is then envisaged for bimetallic catalysts, similarly to what was reported for monometallic Pd/CeO_2_ with the formation of an amorphous Pd–Ce–O layer uniformly surrounding the ceria nanoparticles. The same layer is detected by HRTEM on *0.5Pt*-0.5PdCe M and *0.2Pt*-0.8PdCe M, covering also Pt clusters even if added in the second milling step. On spent bimetallic milled catalysts, the evolution of mushroom-like structures with a PdO foot in contact with the ceria surface covered by a platinum head is observed by HRTEM. These structures, never observed before, evolve at the metal–support interface from a PtPd alloy, which is formed during methane oxidation in dry atmosphere, as inferred from the HRTEM images, XRD, and TPR profiles. The same evolution is different for the impregnated catalyst, for which needle-like arrangements with a PdO stem and a Pt head arise from big PtPd clusters not strictly interacting with the ceria surface and are less effective in promoting the activity and stability of bimetallic catalysts.

## Supporting Information Available

BET and X-ray diffraction profiles of fresh samples; additional TPR experiments; additional HRTEM images; light-off curves for methane oxidation in wet conditions; TPO profiles recorded for 1PdCe M and *0.5Pt*-0.5PdCe M; methane conversion of selected mono- and bimetallic milled samples during TOS dry experiments at 723 K; X-ray diffraction and TPR profiles of *0.5Pt*-0.5PdCe M as prepared, after dry methane oxidation experiment, and after time-on-stream test in wet conditions (PDF)
