# Supplementary material for: Structural Evolution of Bimetallic PtPd/CeO2 Methane Oxidation Catalysts Prepared by Dry Milling
Source: ACS Appl Mater Interfaces. 2021 Jun 2;13(27):31614–23. doi: 10.1021/acsami.1c05050 (PMC8283761; doi:10.1021/acsami.1c05050)
Supplement: Supplementary file 1 — am1c05050_si_001.pdf [file am1c05050_si_001.pdf]

SUPPORTING INFORMATION

Structural evolution of bimetallic PtPd/CeO_2 methane oxidation catalysts prepared by dry milling

Andrea Mussio[a], Maila Danielis[a], Núria J. Divins[b], Jordi Llorca[b],

Sara Colussi[a], Alessandro Trovarelli[a]*

[a] Dipartimento Politecnico, Università degli Studi di Udine and INSTM, via del Cotonificio 108, 33100 Udine, Italy

[b] Institute of Energy Technologies, Department of Chemical Engineering and Barcelona Research Center in Multiscale Science and Engineering, Universitat Politècnica de Catalunya, EEBE, Eduard Maristany 10-14, 08019 Barcelona, Spain

*Corresponding author: sara.colussi@uniud.it

Table S1. BET surface area of all samples

Sample	BET surface area (m^2/g)
CeO_2	3.0
0.8Pt-0.2PdCe M	3.6
0.65Pt-0.35PdCe M	3.6
0.5Pt-0.5PdCe M	4.0
0.2Pt-0.8PdCe M	3.2
0.5PdCe M	3.5
1PdCe M	3.2
*1Pt*Ce M	3.3
0.5Pd-*0.5Pt*Ce M	3.5
0.5Pt-0.5PdCe IWI	3.3

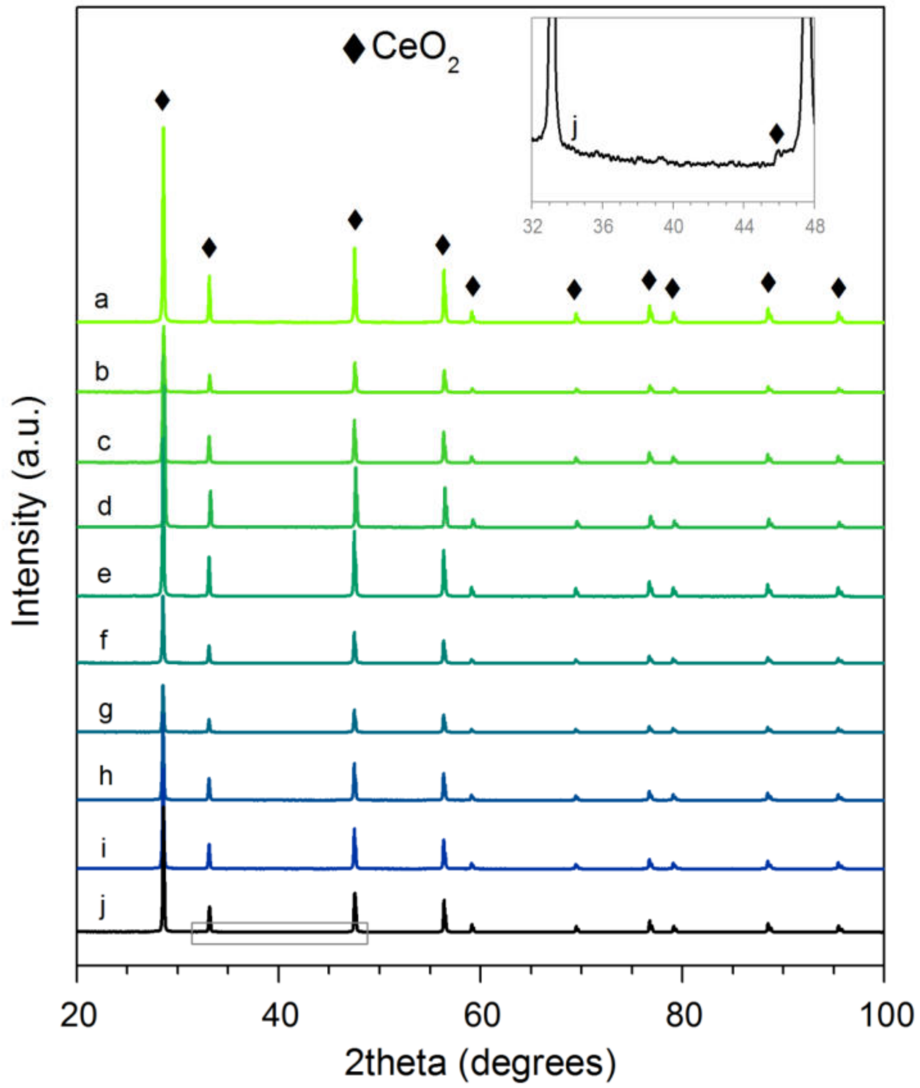

Figure S1: XRD profiles of fresh catalysts in the 20-100° 2θ range: a) 1PdCe M, b) 0.5PdCe M, c) *0.2Pt*-0.8PdCe M, d) *0.5Pt*-0.5PdCe IWI, e) 0.5Pd-*0.5Pt*Ce M, f) *0.5Pt*-0.5PdCe M, g) *0.65Pt*-0.35PdCe M, h) *0.8Pt*-0.2PdCeM, i) *1Pt*Ce M, j) CeO_2. The inset shows an enlargement of the CeO_2 diffraction profile, indicated by the grey square, highlighting the shoulder at 46°.

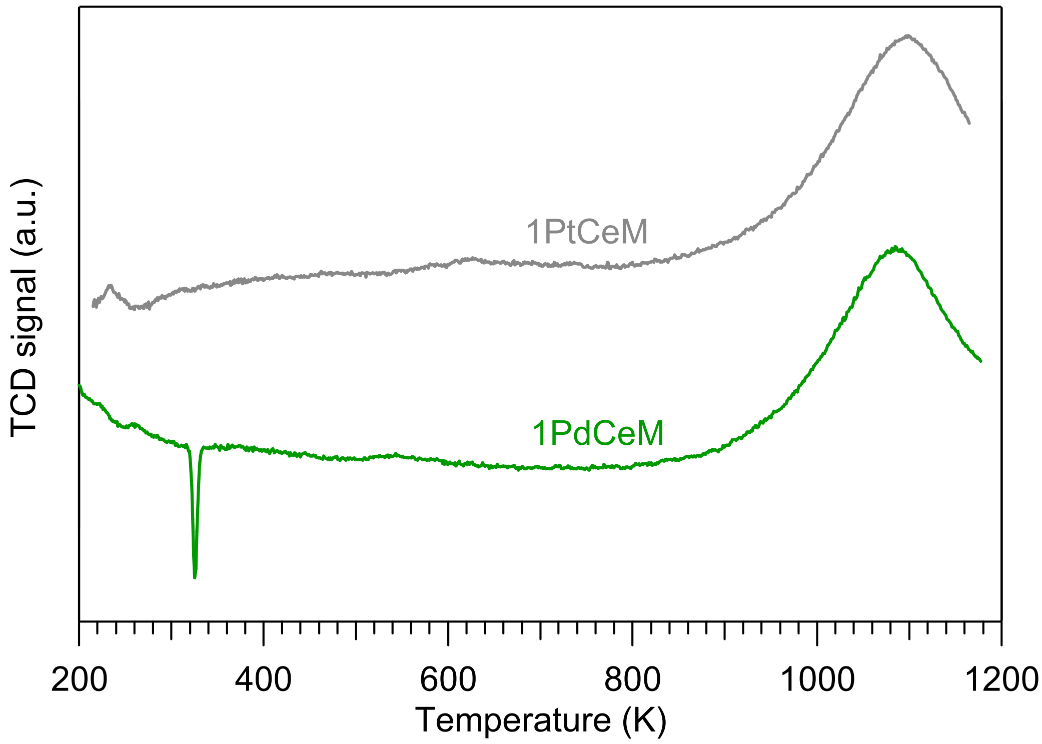

Figure S2: TPR profiles of fresh monometallic samples without pretreatment.

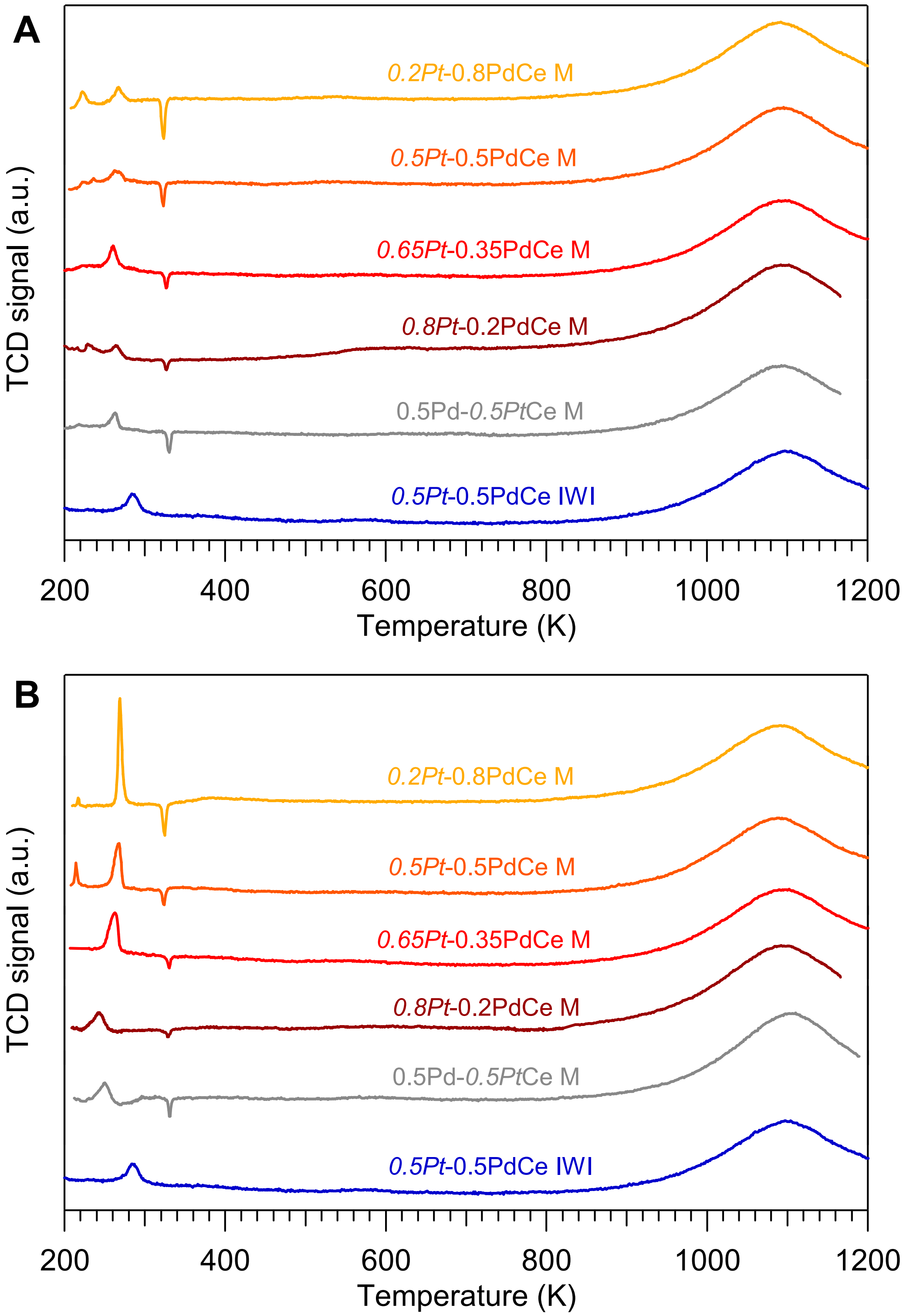

Figure S3: (A) TPR profiles of fresh bimetallic samples; (B) TPR profiles of bimetallic samples after pretreatment in air at 623 K in the whole temperature range.

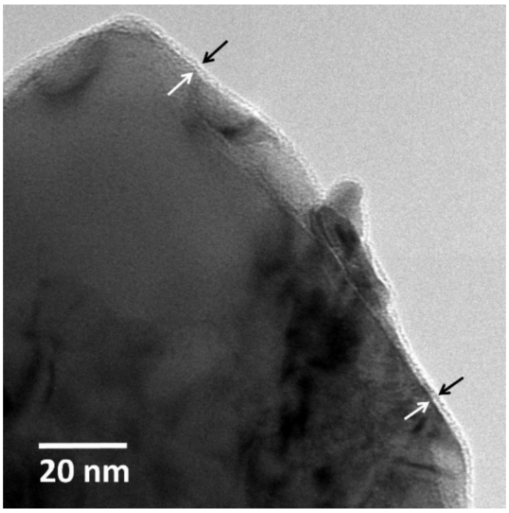

Figure S4: HRTEM image of the amorphous shell on a Pd/CeO_2 monometallic sample prepared by mechanical milling.

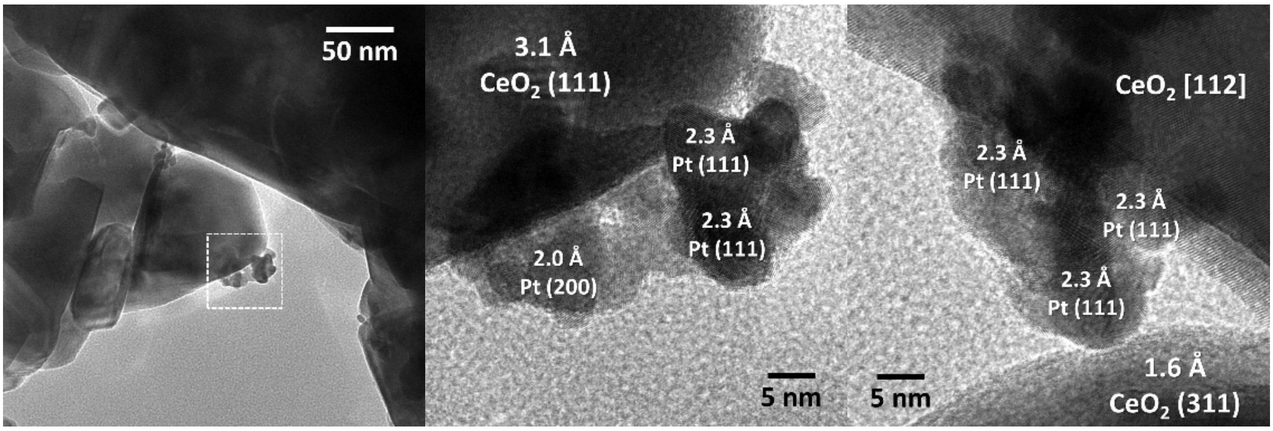

Figure S5: HRTEM images of milled Pt/CeO_2 sample.

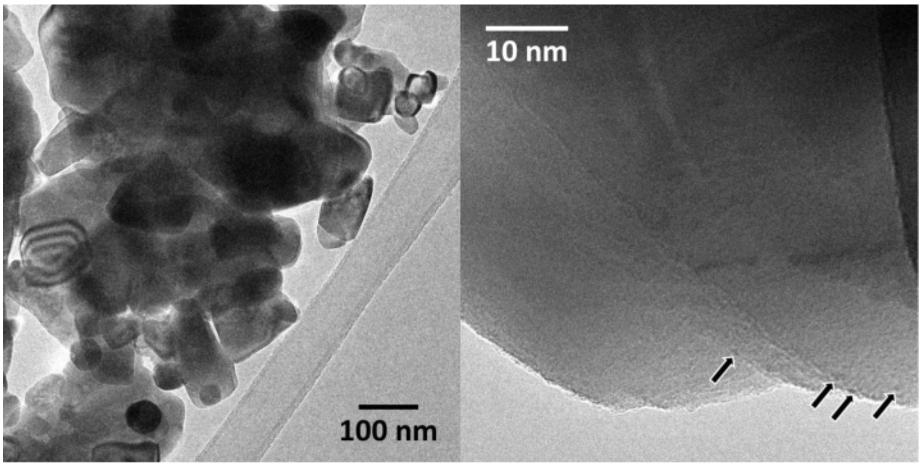

Figure S6: Low magnification (left) and high resolution (right) TEM images of sample *0.5Pt*-0.5PdCe IWI, as prepared.

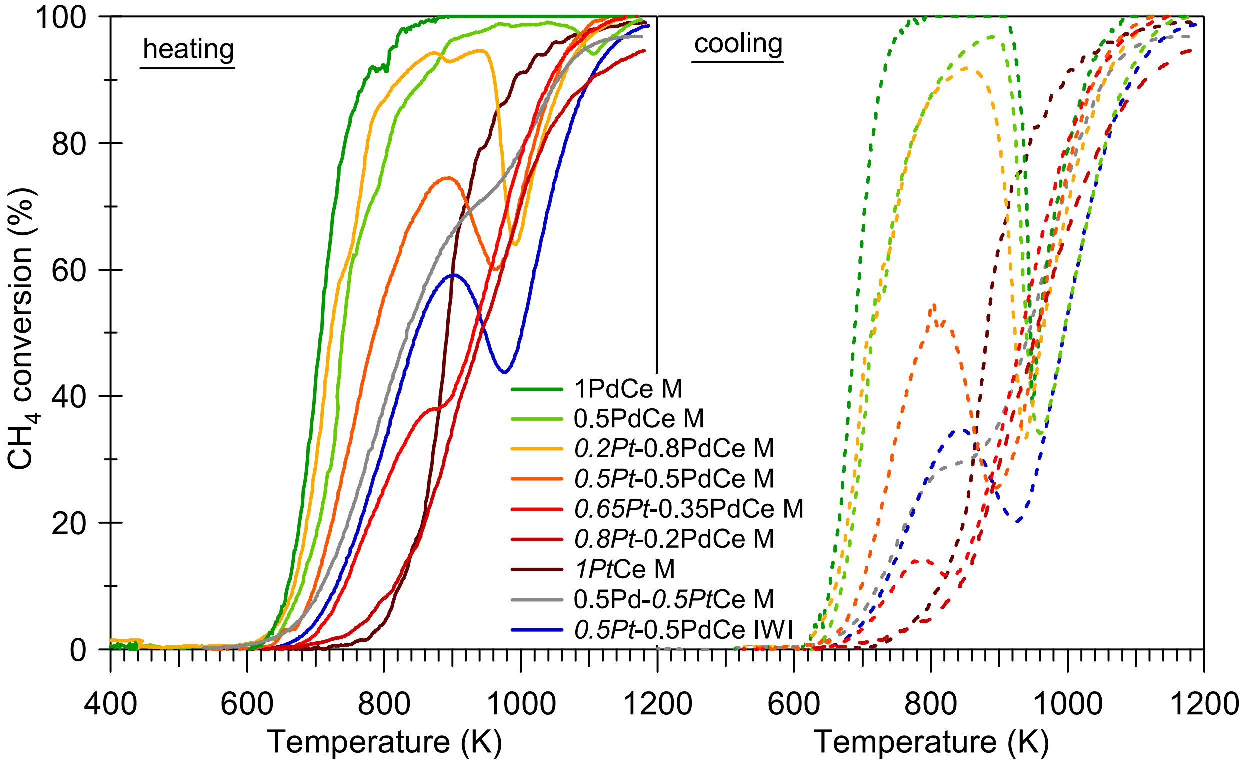

Figure S7: Light-off curves for methane oxidation in wet conditions (0.5 vol% CH_4, 2 vol% O_2, 10 vol% H_2O in He, GHSV ≈ 180000 h^{-1}) for all samples.

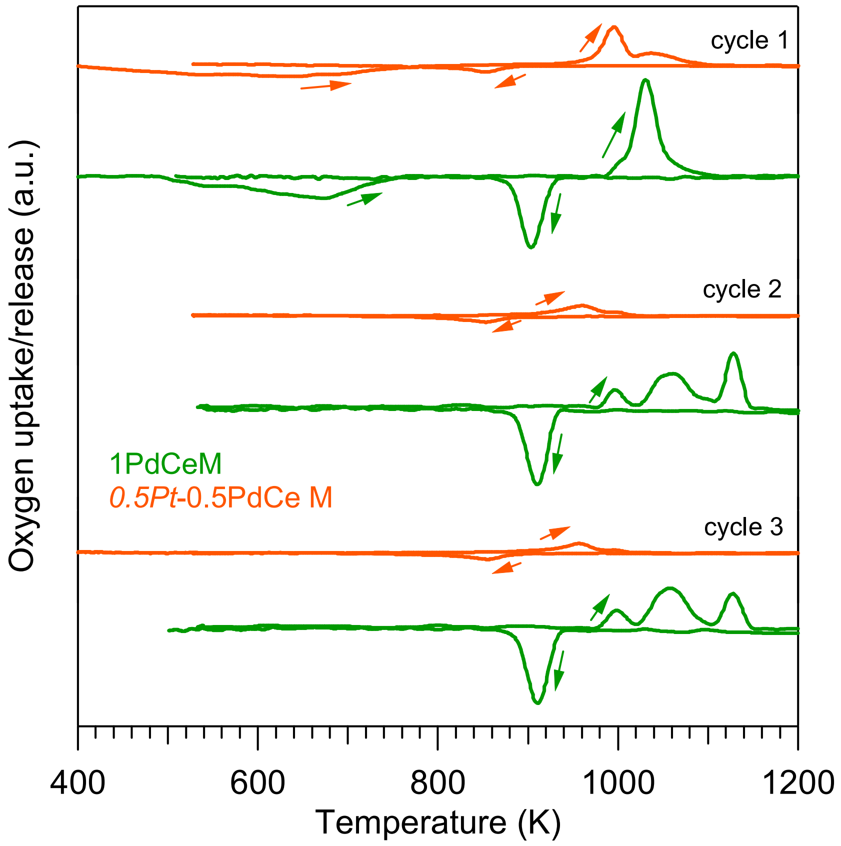

Figure S8: TPO profiles recorded for 1PdCe M and *0.5Pt*-0.5PdCe M, three heating/cooling cycles.

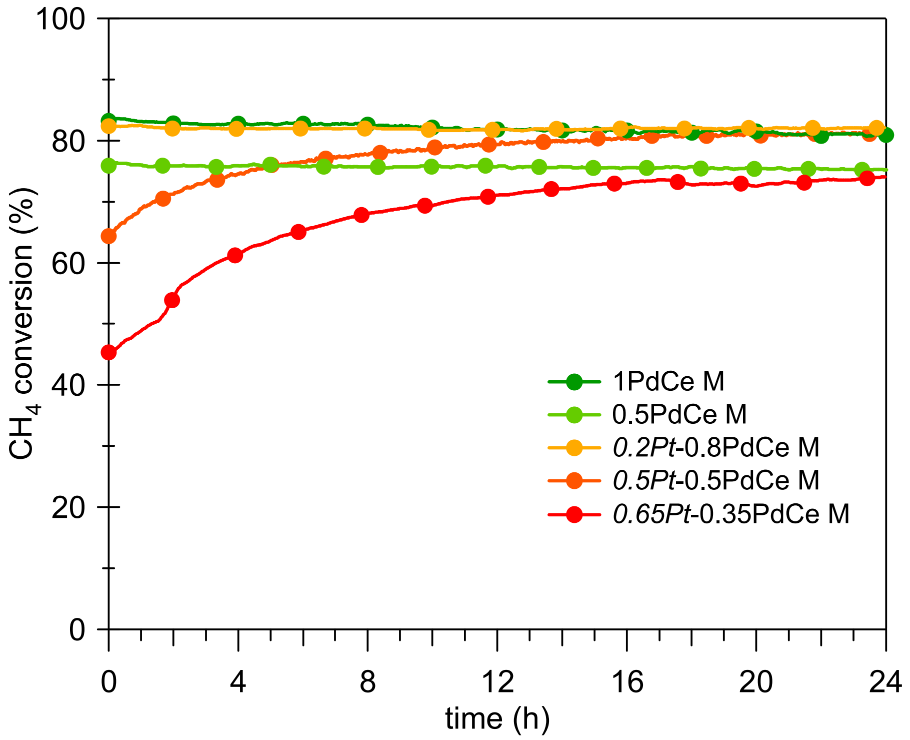

Figure S9: Methane conversion of selected mono- and bimetallic milled samples during TOS dry experiments (0.5% CH_4, 2% O_2 in He) at 723 K.

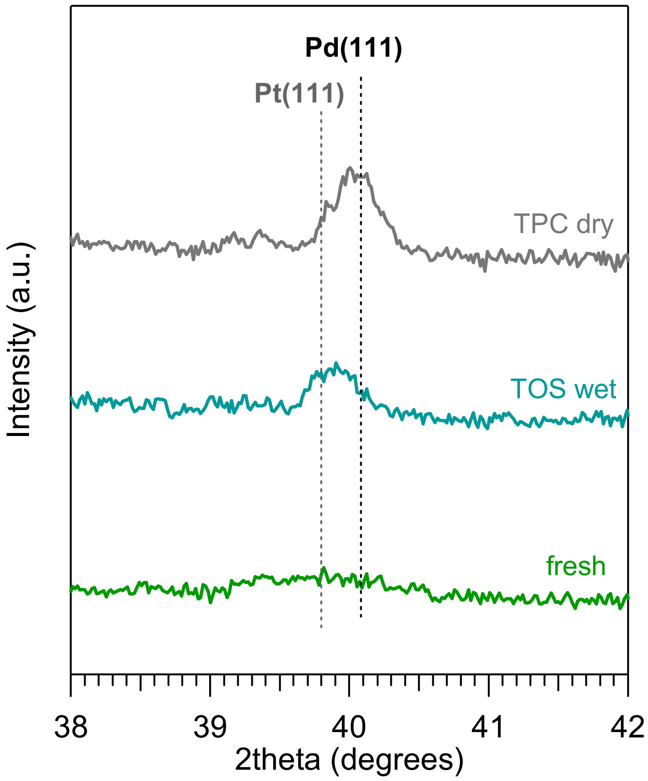

Figure S10: XRD profiles collected on *0.5Pt*-0.5PdCe M as prepared (fresh), after dry methane oxidation experiment (TPC dry) and after Time-On-Stream test in wet conditions (TOS wet).

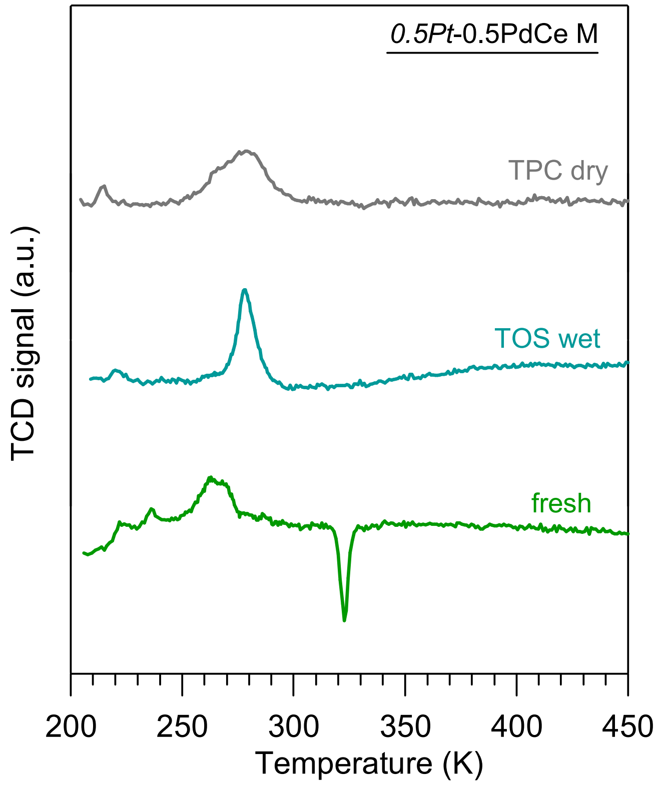

Figure S11: H_2-TPR profiles of *0.5Pt*-0.5PdCe M as prepared (fresh), after dry methane oxidation experiment (TPC dry) and after Time-On-Stream test in wet conditions (TOS wet).

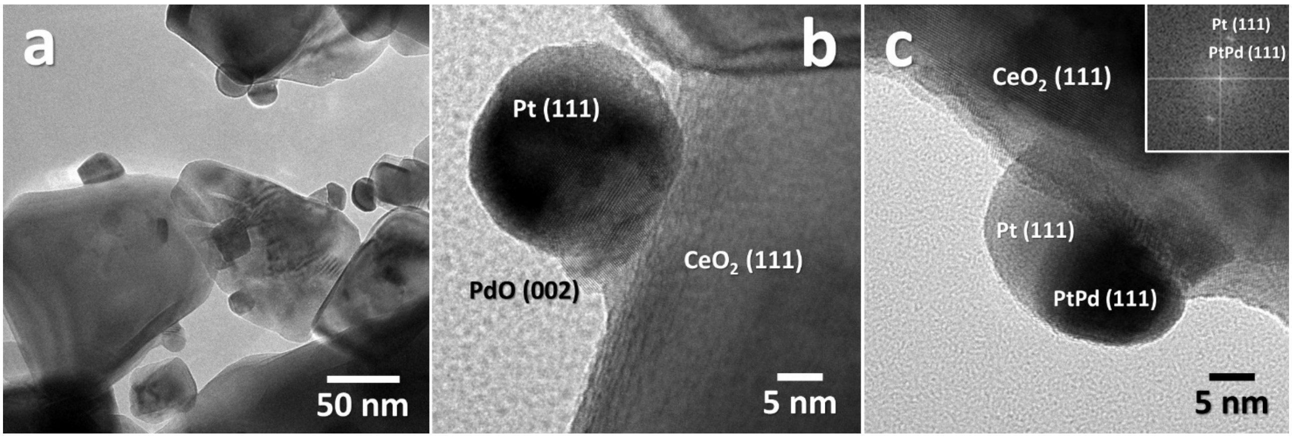

Figure S12: HRTEM images of *1.5Pt*-1.5PdCe M after methane oxidation experiment (TPC) at 923 K.
